# Distributed Averaging Problems of Agriculture Picking Multi-Robot Systems *via* Sampled Control

**DOI:** 10.3389/fpls.2022.898183

**Published:** 2022-07-14

**Authors:** Fengying Ma, Hui Yao, Mingjun Du, Peng Ji, Xiaoan Si

**Affiliations:** School of Information and Automation Engineering, Qilu University of Technology (Shandong Academy of Sciences), Jinan, China

**Keywords:** average consensus, directed communication topologies, distributed protocol, smart agriculture, sampled data

## Abstract

Distributed control of agriculture picking multi-robot systems has been widely used in the field of smart agriculture, this paper aims to explore the distributed averaging problems of agriculture picking multi-robot systems under directed communication topologies by taking advantage of the sampled data. With the algebraic graph theory concepts and the matrix theory, a distributed protocol is proposed based on the nearest sampled neighbor information. It is shown that under the proposed protocol, the states of all agents can be guaranteed to reach average consensus whose value is the averaging of the initial states of all agents. Besides, when considering time-delay, the other distributed protocol is constructed, in which a time margin of the time-delay can be determined simultaneously. The necessary and sufficient consensus results can be developed even though the time delay exists. Simulation results are given to demonstrate the effectiveness of our developed consensus results.

## 1. Introduction

Recently, the issue of smart agriculture has attracted wide attention. Driven by the digital revolution, agriculture has entered a new era of digital and intelligent development (Yang et al., 2013; Alsamhi et al., 2019a; Horng et al., 2019; Fuentes et al., 2021; Li and Chao, 2022; Teng et al., 2022). Smart agriculture is a modern agricultural production mode with information, theoretical knowledge, and hardware equipment as the core elements, and it is an important direction of the development of modern agriculture (Chen and Yang, 2019). Realizing precision agriculture is a goal of smart agriculture (Luo et al., 2016), the present stage of agriculture at a relatively low level of agricultural mechanization, especially in the area of vegetable picking. The traditional way of vegetables is picked manually, which requires a lot of labor during the picking season. Although some plantations have begun to mechanize agricultural picking, the level of automation is low, and vegetable picking is usually carried out by a single mechanical equipment (Brondino et al., 2021), which is inefficient and costly. Therefore, it is urgent to improve the efficiency and mechanization of vegetable picking agriculture.

The emergence of the multi-agent system provides a new trend for the development of smart agriculture. Multi-agent systems are composed of some agents and interactions among agents to solve problems that are impossible for a single agent, which can be applied in many aspects, such as multi-robots (Yu et al., 2020), unmanned aerial vehicles (Lian and Deshmukh, 2006; Alsamhi et al., 2021; Liang et al., 2021), unmanned ground vehicles (Ma et al., 2006), etc. To realize the cooperation between the agents, communication is the fundamental problem, many scholars have carried out a lot of research on communication. In Alsamhi et al. (2019b), the development status of artificial intelligence in the field of the communication among robots is reviewed, and new ideas for its future research directions are provided. In order to better realize the cooperation between robots and ensure effective communication between robots, Alsamhi et al. (2020) fully analyze and discusses robots in different spatial positions through the fusion of machine learning and communication. A sufficient overview is provided for the use of various machine learning techniques in the communication among robots, and it is shown that machine learning plays an important role in improving the communication among robots. In practical application, information communication will be delayed, which may result in the instability of the control system (Seuret et al., 2018). In Zhang et al. (2022), a more general communication mode between delay subsystems and studies of the effect of time delay on the performance of interconnected systems by using the hybrid system theory with memory has been considered. Agriculture picking multi-robot systems consist of multiple robots and interactions among robots, which can be regarded as a kind of multi-agent system. Distributed control of the multi-agent system is also an important issue in the study of multi-agent systems. Cooperative control tasks of multi-agent include clustering, swarming, clustering, formation, tracking, and other tasks. These collective behaviors can all be unified as consensus problems. As the core problem of distributed cooperative control, consensus means that the state values of all agents tend to be the same as time goes to infinity. The average consensus problem is a special consensus problem, which means that the final convergence value of all agents is the average of the initial value (Hu et al., 2020). In smart agriculture, average consensus plays an important role in improving the precision of agriculture. It can better realize the state consensus among agents through the initial state value of each agent, and complete cooperative agricultural tasks among agricultural multi-agents. Particularly, the average consensus of multi-agent systems also has been widely concerned in computer science, energy ecology, social economics, and other fields.

The development of distributed cooperative control has aroused the attention of many researchers in the fields of automatic control. In Olfati-Saber and Murray (2004), the Laplacian potential has been introduced for calculating the difference among agents, with which the distributed control protocol can be induced to ensure the average consensus of multi-agent systems under the undirected graph. The consensus problems have been extended to multi-agent systems whose topologies are switching (Ren and Beard, 2005), in which the consensus objective can be guaranteed if the topologies are joint connected. In the practical application, due to the limitation of speed and bandwidth of network transmission, the problem of time delay certainly exists (Sun and Wang, 2009; Chen et al., 2017; Yan and Huang, 2017). Besides, how to improve the convergence rate should be considered. In Hu et al. (2019), Zou et al. (2019), Dong et al. (2020), and Ran et al. (2020), the distributed control protocols and their convergence analyses have been investigated for finite-time consensus of multi-agent systems.

At present, continuous control protocols are used in most research, so the requirements for network communication are increased. Sampling control can not only reduce the control cost of the system by reducing information transfer redundancy but also improve the robustness of the system (Guan et al., 2012; Ding and Zheng, 2016; Park et al., 2016). Therefore, how to select the appropriate sampling time and sampling mechanism has become a concern of researchers. In Gao et al. (2009), a sampling control protocol is proposed to analyze the consensus of multi-agent systems with fixed and switched topologies, respectively. In Gao and Wang (2011), the consensus of second-order dynamical systems with time-varying topologies is studied by sampling data. The study shows that the system can be consensus by designing appropriate controller gain and the sampling period when the union graph has a spanning tree. To the best of our knowledge, there are quite limited results concerning distributed averaging problems of multi-agent systems, which are suitable for practical applications (e.g., picking multi-robot systems).

Motivated by the above discussions, we aim to reduce information transfer redundancy and control costs of smart agricultural multi-agent systems by sampling control. There are many kinds and complex communication structures in agricultural multi-agent systems. In this article, we explore the distributed averaging problems of the multi-robot systems for vegetable picking agriculture under directed communication topologies. We design a distributed control protocol by taking advantage of the nearest neighbor sampled information for the agriculture picking multi-robot system. With this protocol being used, the necessary and sufficient conditions can be provided for the average consensus objective of the agriculture picking multi-robot system. We further give how to select the sampled period. Besides, in the practical application of multi-agent systems, communication delays may exist. Especially when carrying out agricultural cooperation tasks, information needs to be transmitted between agents. Excessive communication delay will lead to the failure of agricultural multi-agent systems to achieve state consensus and thus fail to complete agricultural tasks. Therefore, we also consider the existence of time-delay among agents. We use the bilinear transformation method to develop the associated convergence analysis, which can simultaneously determine the time margin. It is shown that the agriculture picking multi-robot system can achieve the average consensus if and only if both sampled period and time delay satisfies the appropriate conditions. In addition, simulation examples are carried out to verify the theoretical results. Different from other existing sampling control studies, we carry out an average consensus analysis on picking multi-robot systems with fixed communication topology as a directed graph. The major contributions of this article include:

- the average consensus of agricultural multi-agent systems with fixed communication topology as a digraph is studied, and a distributed control protocol based on sampling information is proposed to ensure the average consensus of the agricultural multi-agent system.
- considering the communication time-delay, another communication protocol is proposed to ensure that the agricultural multi-agent systems can achieve average consensus even with time-delay.
- through the proposed distributed control protocols, the necessary and sufficient conditions for the system to achieve average consensus without time-delay and with time-delay are obtained. Because the average consensus of the system is achieved through the proposed control protocols, the relationship between the initial value and the state value is determined, and the control of the system can be realized more conveniently.

The remainder is outlined below. In Section 2, we introduce some preliminaries for graph and matrix theory. In Section 3, we introduce the problem statement for the distributed averaging of agricultural multi-agent systems for vegetable picking. Section 4 addresses the average consensus of directed the agriculture picking multi-robot system without and with communication time-delay, respectively. Simulation examples are introduced in Section 5. We give comprehensive conclusions in Section 6.

## 2. Preliminaries for Notations and Graph Theory

In this section, some basic concepts related to notations and graph theory are introduced as follows.

We denote $I_{n}=\left\{1,2,\cdots \;,n\right\}$, R^*n*^ is the set of n-dimensional real numbers, R^*n*×*n*^ is a set of real matrices with *n*-dimensional row vectors and *n*-dimensional column vectors, $1_{n}={\left[1,1,\cdots \;,1\right]}^{T}\in {\text{R}}^{n}$, and 0_*n*_ = [0, 0, ⋯ , 0]^*T*^ ∈ R^*n*^, and diag{α_1_, α_2_, ⋯ , α_*n*_} is a diagonal matrix in which diagonal elements are α_1_, α_2_, ⋯ , α_*n*_, zero are the value of non-diagonal elements.

Let G=(V,E,A) represent a directed digraph, where V= {*v*_1_, *v*_2_, ⋯ , *v*_*n*_} is a node set, E⊆V×V is an edge set and $A=\left[a_{ij}\right]\in {\text{R}}^{n\times n}$ with $a_{ij}>0\Leftrightarrow \left(v_{j},v_{i}\right)\in E$ and $a_{ij}=0\Leftrightarrow \left(v_{j},v_{i}\right)\notin E$. Besides, all neighbors of *v*_*i*_ are denoted as $N\left(v_{i}\right)=\left\{v_{j}:\left(v_{j},v_{i}\right)\in E\right\}\left(i\neq j\right)$. An edge $\left(v_{i},v_{j}\right)\in E$ means that the data can be transmitted from the node *v*_*i*_ to the node *v*_*j*_. Let $Q=\left\{\left(v_{i},v_{r_{1}}),\;(v_{r_{2}},v_{r_{3}}),\;\cdots ,\;(v_{r_{n}},v_{j}\right)\right\}$ stand for a directed way from *v*_*i*_ to *v*_*j*_, where *v*_*i*_, *v*_*r*_1__, *v*_*r*_2__, ⋯ , *v*_*r*_*n*__, *v*_*j*_ are different. The digraph G is called strongly connected on condition that G have at least one directed way between any twain different nodes. Let $\Delta =diag\left\{\sum_{j=1}^{n}a_{1j},\sum_{j=1}^{n}a_{2j},\cdots \;,\sum_{j=1}^{n}a_{nj}\right\}$ be the in-degree matrix of G. The Laplacian matrix *L* ∈ R^*n*×*n*^ of G is defined as *L* = Δ − *A*. Benefiting from *L*, we can introduce the following diagonal matrix


$$\begin{array}{l} W=\text{diag}\left\{\text{det}\left(L_{11}\right),\text{det}\left(L_{22}\right),\cdots \;,\text{det}\left(L_{nn}\right)\right\} \end{array}$$

where $L_{ii}\in {\text{R}}^{\left(n-1\right)\times \left(n-1\right)}$ is induced from the Laplacian matrix *L* by deleting its *i*th row and *i*th column, det (*L*_*ii*_) represents the determinant value of the matrix *L*_*ii*_. The Laplacian matrix *L* of a strongly connected digraph G has a zero eigenvalue and all other eigenvalues with positive real parts. It follows from Li and Jia (2009) that if G is strongly connected, then $w_{l}={\left[\text{det}\left(L_{11}\right),\text{det}\left(L_{22}\right),\cdots \;,\text{det}\left(L_{nn}\right)\right]}^{T}$ is the left eigenvector of *L* associated with the zero eigenvalue, i.e., $w_{l}^{T}L=0_{n}^{T}$. With the help of *W*, we can construct a new graph $\bar{G}=\left(V,\bar{E},\bar{A}\right)$, where the element ${\bar{a}}_{ij}$ of $\bar{A}=\left[{\bar{a}}_{ij}\right]$ satisfies


(1) $$\begin{array}{r} {\bar{a}}_{ij}=\frac{det\left(L_{ii}\right)a_{ij}+det\left(L_{jj}\right)a_{ji}}{2},\;\forall i,j\in I_{n}. \end{array}$$

By taking advantage of (1), we can easily obtain


(2) $$\begin{array}{r} \bar{A}=\frac{WA+A^{T}W}{2}. \end{array}$$

With (2), we can establish the relationship between *L* and $\bar{L}$.

**Lemma 1**. *For any digraph G and $\bar{G}$, let L and $\bar{L}$ be the Laplacian matrix of G and $\bar{G}$, respectively. The Laplacian matrices L and $\bar{L}$ satisfy*


(3) $$\begin{array}{r} \bar{L}=\frac{WL+L^{T}W}{2}. \end{array}$$

**proof** We can directly derive this result with the help of (2) and the definition of Laplacian matrices

**Lemma 2**. *Consider a partitioned matrix* $P=\left[\begin{array}{cc} A & B\\ C & D \end{array}\right]\in {\text{R}}^{\left(r+s\right)\times \left(r+s\right)}$, *where A* ∈ R^*r×r*^, *B* ∈ R^*r*×*s*^, *C* ∈ R^*s*×*r*^, *and D* ∈ R^*s*×*s*^.

1. *If A is an invertible matrix, then* |*P*| = |*A*||*D* − *CA*^−1^*B*|;
2. *If D is an invertible matrix, then* |*P*| = |*D*||*A* − *CD*^−1^*B*|.

In the analysis of discrete-time systems, by using a bilinear transformation, the problem of determining Schur stability of a discrete-time system can be transformed into the problem of determining Hurwitz stability of a continuous-time system. Given a polynomial with complex coefficients:


(4) $$\begin{array}{r} g\left(s\right)=\rho_{n}s^{n}+\rho_{n-1}s^{n-1}+\cdots +\rho_{1}s+\rho_{0} \end{array}$$

where $\rho \in \text{C},i\in I_{n}.$ Perform a bilinear transformation $z=\frac{s+1}{s-1}$ on *g*(*s*), a new polynomial is deduced


(5) $$\begin{array}{r} f\left(z\right)={\left(z-1\right)}^{n}g\left(\frac{z+1}{z-1}\right)=\chi_{0}+\chi_{1}z+\cdots +\chi_{n}z^{n} \end{array}$$

where χ_*i*_ = *a*_*i*_ + ι*b*_*i*_, *a*_*i*_, *b*_*i*_ ∈ R, i = 0, 1,…, n. The Hurwitz stability of *f*(*z*) implies the Schur stability of *g*(*s*). Substituting *z* = *wι* into *f*(*z*), we get


(6) $$\begin{array}{r} f\left(\omega \right)=f_{\omega }\left(\omega \right)+\iota f_{\iota }\left(\omega \right) \end{array}$$

where *f*_ω_(ω), *f*_ι_(ω) ∈ R(ω), and


(7) $$\begin{array}{r} f_{\omega }\left(\omega \right)=a_{0}-b_{1}\omega -a_{2}\omega^{2}+b_{3}\omega^{3}+a_{4}\omega^{4}-\cdots \end{array}$$

(8) $$\begin{array}{r} f_{\iota }\left(\omega \right)=b_{0}+a_{1}\omega -b_{2}\omega^{2}-a_{3}\omega^{3}+b_{4}\omega^{4}-\cdots \end{array}$$

*f*_ω_(ω) and *f*_ι_(ω) constituent interlaced polynomial, to determine whether *f*(*z*) Hurwitz stable, the Hermite-Biehler theorem is given as follows

**Lemma 3**. *(Ogata, 1995) The polynomial f*(*z*) *is Hurwitz stability if and only if the related pair f*_ω_(ω), *f*_ι_(ω) *is interlaced, and* $f_{\omega }\left(0\right){f_{\iota }}^{\prime }\left(0\right)-f_{\omega }^{\prime }\left(0\right)f_{\iota }\left(0\right)>0$.

## 3. Problem Statements

In the picking process of vegetables, such as cucumbers, a device with multiple mechanical arms is used to pick the vegetables. In the actual picking process, multiple mechanical arms pick vegetables at the same time and then put vegetables into the picking robot which runs on a specific track. To accurately collect vegetables picked by mechanical arms, the corresponding collection robots should achieve position states consensus, to better pick and collect vegetables. To solve the problem, a consensus analysis of the robot's position state is needed. In this article, we treat each robot as an intelligent agent and all agents constitute an agriculture multi-agent system. To analyze the consensus of the agriculture multi-agent system, we use a digraph G=(V, E, *A*) of the agricultural multi-agent system to denote the communication topology of the agriculture picking multi-robot system, in which the set of all agents can be described by V, and the relationships among agents can be represented by E and *A*. Let $x_{i}\in {\text{R}}^{n}$ be the position state of agent *v*_*i*_ and $x(t)={\left[x_{1}(t),\;x_{2}(t),\;\cdots \;,\;x_{n}(t)\right]}^{T}\in {\text{R}}^{n}$ denote the state vector. Every agent has the following dynamics


(9) $$\begin{array}{l} {ẋ}_{i}\left(t\right)=u_{i}\left(t\right),\;\forall i\in I_{n} \end{array}$$

where *u*_*i*_ is the control protocol to be designed.

Generally, the decision value of the agriculture multi-agent system not only depends on the topological structure but also on the initial states. However, the average-consensus problems only rely on the initial states and have no relation to the topological structure. That is to say, for random initial states *x*_*i*_(0), $\forall i\in I_{n}$, the average consensus of the system (9) can be reached if


(10) $$\begin{array}{l} \underset{t\to \infty }{\lim}x_{i}\left(t\right)=\frac{1}{n}\overset{n}{\underset{i=1}{\sum }}x_{i}\left(0\right),\forall i\in I_{n}. \end{array}$$

The agricultural multi-agent system (9) can reach average consensus means that we can infer the final position of agents from the initial position, so the control difficulty is reduced, and the controllability of the agriculture multi-agent system (9) is improved.

Since continuous control will increase the communication burden of agricultural multi-agent systems, in order to prevent information redundancy and reduce the cost of systems, we use sampling data to complete the distributed control of the agricultural multi-agent system. The sampling control can improve the robustness of the picking robot system. In what follows, the purpose of this article is to design a distributed control protocol so that the agricultural multi-agent system (9) under the strongly connected digraph G accomplishes the average consensus objective *via* sampled information. Besides, when considering the communication time-delay, we further explore how to develop the time margin of the communication time-delay.

## 4. Main Results

In this section, we investigate the average consensus problems of the agricultural multi-agent system (9) whose communication topologies are directed. Besides, information needs to be transmitted between agents, and the excessive communication time-delay will cause the oscillation or divergence of agricultural multi-agent systems so that the robots in agriculture cannot achieve position state consensus, which means that the robots cannot accurately load the picked vegetables. Thus, we further explore the average consensus problems of agricultural multi-agent systems when there exist communication time-delays among agents.

### 4.1. Distributed Control Protocol Without Time-Delay

In this subsection, to reduce the communication cost of smart agricultural multi-agent systems, we aim to solve the average consensus problems of the agriculture multi-agent system by taking advantage of sampled data. Toward this end, we introduce a distributed control protocol by employing the sampled data as follows:


(11) $$\begin{array}{r} u_{i}\left(t\right)=\underset{j\in N\left(i\right)}{\sum }{\bar{a}}_{ij}\left(x_{j}\left(kp\right)-x_{i}\left(kp\right)\right),t\in \left[kp,kp+p\right),\\ \forall i,j\in I_{n},k=0,1,2,\cdots \end{array}$$

where *p* is the sampled period. Based on $\bar{L}$, we can rewrite (9) and (11) as a compact form


(12) $$\begin{array}{l} x\left(kp+p\right)=\bar{\psi }x\left(kp\right),k=0,1,2,\cdots \end{array}$$

where $\bar{\psi }=I-p\bar{L}$.

In the following, we explore the convergence problems of the system (12). We propose a tree transformation for the system (12). We first introduce a series of states as follows:


(13) $$\begin{array}{l} y_{1}\left(kp\right)=x_{1}\left(kp\right)\\ y_{2}\left(kp\right)=x_{1}\left(kp\right)-x_{2}\left(kp\right)\\ y_{3}\left(kp\right)=x_{1}\left(kp\right)-x_{3}\left(kp\right)\\ \;\vdots \\ y_{n}\left(kp\right)=x_{1}\left(kp\right)-x_{n}\left(kp\right). \end{array}$$

Denote


$$\begin{array}{l} Q=\left[\begin{array}{ccccc} 1 & 0 & 0 & \cdots & 0\\ 1 & -1 & 0 & \cdots & 0\\ 1 & 0 & -1 & \cdots & 0\\ \vdots & \vdots & \vdots & \ddots & \vdots \\ 1 & 0 & 0 & \cdots & -1 \end{array}\right]=\left[\begin{array}{c} C\in {\text{R}}^{1\times n}\\ E\in {\text{R}}^{\left(n-1\right)\times n} \end{array}\right] \end{array}$$

and the inverse matrix of *Q* is given by


$$\begin{array}{l} Q^{-1}=\left[\begin{array}{ccccc} 1 & 0 & 0 & \cdots & 0\\ 1 & -1 & 0 & \cdots & 0\\ 1 & 0 & -1 & \cdots & 0\\ \vdots & \vdots & \vdots & \ddots & \vdots \\ 1 & 0 & 0 & \cdots & -1 \end{array}\right]=\left[\begin{array}{cc} w_{r}\in {\text{R}}^{n\times 1} & F\in {\text{R}}^{n\times \left(n-1\right)} \end{array}\right]. \end{array}$$

With the help of *Q*, the states *y*_1_(*kp*), *y*_2_(*kp*), ⋯ , *y*_*n*_(*kp*) are defined by


$$\begin{array}{l} y\left(kp\right)≜\left[\begin{array}{c} y_{1}\left(kp\right)\\ y_{2}\left(kp\right)\\ \vdots \\ y_{n}\left(kp\right) \end{array}\right]=Qx\left(kp\right). \end{array}$$

Substituting *y*(*kp* + *p*) = *Qx*(*kp* + *p*) and *y*(*kp*) = *Qx*(*kp*) into the system (12) leads to


(14) $$\begin{array}{l} y\left(kp+p\right)=\left(I-pH\right)y\left(kp\right) \end{array}$$

where $H=Q\bar{L}Q^{-1}=\left[\begin{array}{cc} 0 & C\bar{L}F\\ 0_{n-1} & E\bar{L}F \end{array}\right].$ We denote $ŷ\left(kp\right)={\left[y_{2}\left(kp\right),y_{3}\left(kp\right),\cdots \;,y_{n}\left(kp\right)\right]}^{T}$ and the system (14) can be divided into two subsystems:


(15) $$\begin{array}{l} y_{1}\left(kp+p\right)=y_{1}\left(kp\right)-pC\bar{L}Fŷ\left(kp\right) \end{array}$$

and


(16) $$\begin{array}{l} ŷ\left(kp+p\right)=\left(I-pE\bar{L}F\right)ŷ\left(kp\right). \end{array}$$

From (16), we can easily see that the reduced system (16) achieving stability implies the consensus of the system (12). Hence, the consensus problem of (12) turns into the asymptotic stability problem of a reduced-order system (16).

With protocol (11) being employed, the average consensus results can be obtained in the following theorem.

**Theorem 1**. *For the system (9) whose communication topology is the strongly connected digraph* G, *let the distributed control protocol (13) be used. Then, the system (9) can achieve the average consensus objective if and only if the following condition holds*.


(17) $$\begin{array}{l} 0<p<\text{mi}{\text{n}}_{\lambda_{i}\neq 0}\frac{2}{\lambda_{i}},\;\forall i\in I_{n}. \end{array}$$

*Proof*. The digraph G is strongly connected, its Laplacian matrix *L* has a zero eigenvalue and *n* − 1 non-zero eigenvalue with positive real parts. It follows from Lemma1 that $\bar{L}$ is a symmetric matrix. The eigenvalues of $\bar{L}$ contain a zero eigenvalue and *n* − 1 positive real numbers. Based on our defined *H*, we realize that the eigenvalues of $E\bar{L}F$ are positive real numbers. To ensure the reduced-order system (16) is stable, the condition $\rho \left(I-pE\bar{L}F\right)<1$. Next, we target at exploring how to ensure $\rho \left(I-pE\bar{L}F\right)<1$ by picking up the sampled period *p*.

We introduce an inverse matrix *T* such that


(18) $$\begin{array}{l} T^{-1}E\bar{L}FT=\Lambda =\left[\begin{array}{cccc} \lambda_{2} & * & \; & \;\\ \; & \lambda_{3} & \ddots & \;\\ \; & \; & \ddots & *\\ \; & \; & \; & \lambda_{n} \end{array}\right] \end{array}$$

where λ_2_, λ_3_, ⋯ , λ_*n*_ are the nonzero eigenvalues of $\bar{L}$ and the elements * maybe 0 or 1. Then, employing ỹ(*kp* + *p*) = *P*^−1^ŷ(*kp* + *p*), ỹ(*kp*) = *P*^−1^ŷ(*kp*), we can convert the reduced-order system (16) into


(19) $$\begin{array}{l} ỹ\left(kp+p\right)=\left(I-p\Lambda \right)ỹ\left(kp\right). \end{array}$$

The stability of the systems (16) and (19) are equivalent. We can further induce


(20) $$\begin{array}{l} \rho \left(I-p\Lambda \right)<1\Leftrightarrow |1-p\lambda_{i}|<1\text{ for all eigenvalues of }\bar{L}. \end{array}$$

Therefore, ρ(*I* − *p*Λ) < 1 holds if and only if *p* meets the condition (17) holds. Based on the condition (17), the reduced system (16) can reach stability, which denotes that the system (9) is able to achieve the consensus.

Next, we calculate the convergence value of the dynamic system (9). Let *w*_*r*_ and *w*_*l*_ denote the right eigenvector and left eigenvector of $\bar{L}$ associated with its eigenvalue 0, respectively, which satisfy $w_{l}^{T}w_{r}=1$. Correspondingly, we can easily obtain that *w*_*r*_ and *w*_*l*_ are also the right eigenvector and left eigenvector of $\bar{\psi }=I-p\bar{L}$ associated with the eigenvalue 1. Since the digraph G is strongly connected, we can develop that $\bar{G}$ is undirected and connected. Without loss of generality, we select *w*_*r*_ and *w*_*l*_ that satisfy $w_{r}=w_{l}=\frac{1}{\sqrt{n}}1_{n}$. With (9), we can deduce


$$\begin{array}{l} x\left(kp+p\right)=\bar{\psi }x\left(kp\right)\\ \;={\bar{\psi }}^{k+1}x\left(0\right). \end{array}$$

Because $\bar{\psi }$ has an eigenvalue 1 and *n* − 1 eigenvalues whose module is less than 1. Hence, we can develop


$$\begin{array}{l} \underset{k\to \infty }{\lim}x\left(kp\right)=\underset{k\to \infty }{\lim}{\bar{\psi }}^{k}x\left(0\right)\\ \;=w_{r}w_{l}^{T}x\left(0\right)=\frac{1}{n}\overset{n}{\underset{i=1}{\sum }}x_{i}\left(0\right) \end{array}$$

which implies the system (9) can achieve the average consensus objective *via* sampled control. The sampled control can be used to reduce the communication cost of the agricultural multi-agent system (9). We complete this proof.

### 4.2. Distributed Control Problems With Time-Delay

It is shown that under the proposed protocol without time-delay, the states of all agents can be guaranteed to reach a consensus whose value is the averaging of the initial states of all agents. However, in the practical application of agriculture multi-agent systems, it may suffer from the effect of communication time-delay. Especially in the completion of agricultural cooperative tasks, information needs to be transmitted between agents, and the excessive communication delay will cause the oscillation or divergence of multi-agent systems, so the time-delay problem needs to be considered. When considering the existing time-delay τ which is less than 1 sampling period, the distributed control protocol is constructed by


(21) $$\begin{array}{l} u_{i}\left(t\right)=\{\begin{array}{l} \underset{j\in N\left(i\right)}{\sum }{\bar{a}}_{ij}\left(x_{j}\left(kp-p\right)-x_{i}\left(kp-p\right)\right),t\in \left[kp,kp+\tau \right)\\ \underset{j\in N\left(i\right)}{\sum }{\bar{a}}_{ij}\left(x_{j}\left(kp\right)-x_{i}\left(kp\right)\right),t\in \left[kp+\tau ,kp+p\right) \end{array}\\ \;\forall i,j\in I_{n},k=0,1,2,\cdots \;,\;0<\tau <p. \end{array}$$

We rewrite (9) and (21) as follows


(22) $$\begin{array}{l} \left[\begin{array}{c} x\left(kp+p\right)\\ x\left(kp\right) \end{array}\right]=\phi \left[\begin{array}{c} x\left(kp\right)\\ x\left(kp-p\right) \end{array}\right],k=0,1,2,\cdots \end{array}$$

where


(23) $$\begin{array}{l} \phi =\left[\begin{array}{cc} I_{n}-\left(p-\tau \right)\bar{L} & -\tau \bar{L}\\ I_{n} & 0 \end{array}\right]. \end{array}$$

We can deduce from (22) and (23) that


(24) $$\begin{array}{l} x\left(kp+p\right)=\left[I_{n}-\left(p-\tau \right)\bar{L}\right]x\left(kp\right)-\tau \bar{L}x\left(kp-p\right),\;k=0,1,2,\cdots \end{array}$$

In the following, we also use the tree transformation for the system (22), the consensus problem of (22) turns into the asymptotic stability problem of a reduced-order system.

Substituting *y*(*kp* + *p*) = *Qx*(*kp* + *p*), *y*(*kp*) = *Qx*(*kp*), and *y*(*kp* − *p*) = *Qx*(*kp* − *p*) into the system (22) leads to


(25) $$\begin{array}{l} \left[\begin{array}{c} y\left(kp+p\right)\\ y\left(kp\right) \end{array}\right]=\left[\begin{array}{cc} I_{n}-\left(p-\tau \right)H & -\tau H\\ I_{n} & 0_{n\times n} \end{array}\right]\left[\begin{array}{c} y\left(kp\right)\\ y\left(kp-p\right) \end{array}\right] \end{array}$$

where $H=Q\bar{L}Q^{-1}=\left[\begin{array}{cc} 0 & C\bar{L}F\\ 0_{n-1} & E\bar{L}F \end{array}\right].$

We denote


$$\begin{array}{l} ŷ\left(kp\right)={\left[y_{2}\left(kp\right),y_{3}\left(kp\right),\cdots \;,y_{n}\left(kp\right)\right]}^{T},\\ ŷ\left(kp-p\right)={\left[y_{2}\left(kp-p\right),y_{3}\left(kp-p\right),\cdots \;,y_{n}\left(kp-p\right)\right]}^{T} \end{array}$$

and the system (25) can be divided into two subsystems:


(26) $$\begin{array}{r} \left[\begin{array}{c} y_{1}\left(kp+p\right)\\ y_{1}\left(kp\right) \end{array}\right]=\left[\begin{array}{cc} 1 & 0\\ 1 & 0 \end{array}\right]\left[\begin{array}{c} y_{1}\left(kp\right)\\ y_{1}\left(kp-p\right) \end{array}\right]\\ -\left[\begin{array}{cc} \left(p-\tau \right)C\bar{L}F & \tau C\bar{L}F\\ 0 & 0 \end{array}\right]\left[\begin{array}{c} ŷ\left(kp\right)\\ ŷ\left(kp-p\right) \end{array}\right] \end{array}$$

and


(27) $$\begin{array}{r} \left[\begin{array}{c} ŷ\left(kp+p\right)\\ ŷ\left(kp\right) \end{array}\right]=\left[\begin{array}{cc} I_{n-1}-\left(p-\tau \right)E\bar{L}F & -\tau E\bar{L}F\\ I_{n-1} & 0_{\left(n-1\right)\times \left(n-1\right)} \end{array}\right]\\ \left[\begin{array}{c} ŷ\left(kp+p\right)\\ ŷ\left(kp-p\right) \end{array}\right]. \end{array}$$

From (27), we can easily see that the reduced system (27) achieving stability implies the consensus of the system (9). With the protocol (21) being employed, the average consensus results can be obtained in the theorem below.

**Theorem 2**. *For the system (9) whose communication topology is the strongly connected digraph* G, *let the distributed control protocol (21) be used. Then, the system (9) can achieve the average consensus objective if and only if the following condition holds*.


(28) $$\begin{array}{l} \tau <\text{mi}{\text{n}}_{\lambda_{i}\neq 0}\frac{1}{\lambda_{i}},\;0<p<\text{mi}{\text{n}}_{\lambda_{i}\neq 0}\frac{2}{\lambda_{i}}+2\tau ,\;\forall i\in I_{n}. \end{array}$$

*Proof*. Since the digraph G is strongly connected, its Laplacian matrix *L* has a zero eigenvalue and *n* − 1 non-zero eigenvalue with positive real parts. It follows from Lemma1 that $\bar{L}$ is a symmetric matrix.

We introduce an inverse matrix *T* such that


(29) $$\begin{array}{l} T^{-1}E\bar{L}FT=\Lambda =\left[\begin{array}{cccc} \lambda_{2} & * & \; & \;\\ \; & \lambda_{3} & \ddots & \;\\ \; & \; & \ddots & *\\ \; & \; & \; & \lambda_{n} \end{array}\right] \end{array}$$

where λ_2_, λ_3_, ⋯ , λ_*n*_ are the nonzero eigenvalues of $\bar{L}$ and the elements * may be 0 or 1. Then, employing ỹ(*kp* + *p*) = *T*^−1^ŷ(*kp* + *p*), ỹ(*kp*) = *T*^−1^ŷ(*kp*), and ỹ(*kp* − *p*) = *T*^−1^ŷ(*kp* − *p*) we can convert the reduced-order system (27) into


(30) $$\begin{array}{l} \left[\begin{array}{c} ỹ\left(kp+p\right)\\ ỹ\left(kp\right) \end{array}\right]=\zeta \left[\begin{array}{c} ỹ\left(kp+p\right)\\ ỹ\left(kp-p\right) \end{array}\right] \end{array}$$

where


(31) $$\begin{array}{l} \zeta =\left[\begin{array}{cc} I_{n-1}-\left(p-\tau \right)\Lambda & -\tau \Lambda \\ I_{n-1} & 0_{\left(n-1\right)\times \left(n-1\right)} \end{array}\right]. \end{array}$$

The stability of the systems (27) and (30) are equivalent. The characteristic polynomial of ζ is given by


(32) $$\begin{array}{l} det\left(sI_{2n-2}-\zeta \right)=\left|\begin{array}{cc} sI_{n-1}-\left[I_{n-1}-\left(p-\tau \right)\Lambda \right] & \tau \Lambda \\ -I_{n-1} & sI_{n-1} \end{array}\right|. \end{array}$$

From (32), we can easily obtain that ξ is a partitioned matrix. It follows from Lemma 2 that


(33) $$\begin{array}{l} \left|\xi |=|sI_{n-1}||sI_{n-1}-\left[I_{n-1}-\left(p-\tau \right)\Lambda \right]+I_{n-1}{\left(sI_{n-1}\right)}^{-1}\tau \Lambda \right|\\ \;=\left|\begin{array}{ccc} s & \; & \;\\ \; & \ddots & \;\\ \; & \; & s \end{array}\right|\left|\begin{array}{ccc} s-\left[1-\left(p-\tau \right)\lambda_{2}\right]+\frac{1}{s}\tau \lambda_{2} & \; & \;\\ \; & \ddots & \;\\ \; & \; & s-\left[1-\left(p-\tau \right)\lambda_{n}+\frac{1}{s}\tau \lambda_{n}\right] \end{array}\right|\\ \;=\overset{n}{\underset{i=2}{\prod }}\left[s^{2}-\left(1-p\lambda_{i}+\tau \lambda_{i}\right)s+\tau \lambda_{i}\right]\\ \;=\overset{n}{\underset{i=2}{\prod }}g_{i}\left(s\right). \end{array}$$

Then, by applying bilinear transformation $s=\frac{z+1}{z-1}$, we have


(34) $$\begin{array}{l} f_{i}\left(z\right)=p\lambda_{i}z^{2}+2\left(1-\tau \lambda_{i}\right)z+\left(p-2\tau \right)\lambda_{i}+2 \end{array}$$

We can prove the polynomial *g*_*i*_(*s*) is Schur stable by making sure that the polynomial (34) is Hurwitz stable. Let *z* = ωι, we can further deduce


(35) $$\begin{array}{l} f_{i}\left(\omega \right)=-p\lambda_{i}\omega^{2}+2\left(1-\tau \lambda_{i}\right)\omega \iota +\left(p-2\tau \right)\lambda_{i}+2. \end{array}$$

The real part and imaginary part of (35) are given by


(36) $$\begin{array}{l} f_{\omega }\left(\omega \right)=-p\lambda_{i}\omega^{2}+\left(p-2\tau \right)\lambda_{i}+2 \end{array}$$

and


(37) $$\begin{array}{l} f_{\iota }\left(\omega \right)=2\left(1-\tau \lambda_{i}\right)\omega \iota \end{array}$$

The polynomial (35) is Hurwitz stable if and only if the following conditions hold.

C1) *f*_ω_(ω) = 0 has two distinct roots γ_1_ < γ_2_.
C2) The real root γ_3_ of *f*_ι_ = 0 satisfies γ_1_ < γ_3_ < γ_2_.
C3) $f_{\omega }\left(0\right)f_{\iota }^{\prime }\left(0\right)-f_{\omega }^{\prime }\left(0\right)f_{\iota }\left(0\right)>0$.

The condition C1) can be guaranteed by


(38) $$\begin{array}{l} \Delta_{f_{\omega }}=4\left(p\lambda_{i}\right)\left[2-\left(p-2\tau \right)\lambda_{i}\right]>0. \end{array}$$

Noticing *p* > 0 and λ_*i*_(*i* = 2, 3, …, *N*). Based on (38), we further induce


(39) $$\begin{array}{l} 0<p<\text{mi}{\text{n}}_{\lambda_{i}\neq 0}\frac{2}{\lambda_{i}}+2\tau ,\;\forall i\in I_{n}. \end{array}$$

If Δ_*f*_ω__ > 0, we can calculate two roots γ_1_ and γ_2_ of *f*_ω_(ω) = 0 as follows:


$$\begin{array}{l} \gamma_{1}=-\frac{\sqrt{\Delta_{f_{\omega }}}}{2p\lambda_{i}},\gamma_{2}=\frac{\sqrt{\Delta_{f_{\omega }}}}{2p\lambda_{i}}. \end{array}$$

Based on *f*_ι_ = 0, we can get its root γ_3_ = 0. The condition C2) is naturally satisfied. It is mainly because that γ_1_ < 0 and γ_2_ > 0 hold. Motivated by the condition C3), we can deduce


(40) $$\begin{array}{l} \left[2-\left(h-2\tau \right)\lambda_{i}\right]\left(1-\tau \lambda_{i}\right)>0. \end{array}$$

With (39) and (40), we can develop that the system (35) is Hurwitz stable if and only if *p* and τ meet (28). Therefore, we can develop that the reduced system (27) achieves asymptotic stability. It denotes that the system (22) can achieve the consensus objective.

Next, we calculate the convergence value of the system (22). One of the eigenvalues of $\bar{L}$ is 0, Correspondingly, we can infer that 1 are the eigenvalues of ϕ and the module value of other eigenvalues is less than 1. Because $\bar{G}$ is undirected and connected, we can pick up the right eigenvector and the left eigenvector of the matrix ϕ corresponding to the eigenvalue 1 as follows:


$$\begin{array}{l} w_{r}=w_{l}=\frac{1}{\sqrt{n}}1_{n} \end{array}$$

which satisfy $w_{l}^{T}w_{r}=1$. According to (24), we have


$$\begin{array}{l} \left[\begin{array}{c} x\left(kp+p\right)\\ x\left(kp\right) \end{array}\right]=\phi \left[\begin{array}{c} x\left(kp\right)\\ x\left(kp-p\right) \end{array}\right]=\phi^{k}\left[\begin{array}{c} x\left(0\right)\\ x\left(0\right) \end{array}\right]. \end{array}$$

Because ϕ all the other eigenvalues are in the unit circle. Hence, we can develop


$$\begin{array}{l} \underset{k\to \infty }{\lim}\left[\begin{array}{c} x\left(kp+p\right)\\ x\left(kp\right) \end{array}\right]=\left[\begin{array}{cc} w_{r}w_{l}^{T} & 0\\ w_{r}w_{l}^{T} & 0 \end{array}\right]\left[\begin{array}{c} x\left(0\right)\\ x\left(0\right) \end{array}\right]=\left[\begin{array}{c} \frac{1}{n}\sum_{i=1}^{n}x_{i}\left(0\right)\\ \frac{1}{n}\sum_{i=1}^{n}x_{i}\left(0\right) \end{array}\right] \end{array}$$

which implies that the system (9) can achieve the average consensus even if the sampled instance *p* and the communication time-delay τ satisfy the condition (28). This proof is complete.

### 4.3. Analysis and Comparison of Protocols

We compare and analyze the condition of achieving the average consensus of the agricultural multi-agent system without time-delay and with time-delay. The protocols in different cases and the conditions for achieving average consensus in agricultural multi-agent systems are given in Table 1, where λ_*i*_ ≠ 0 and $\;\forall i,j\in I_{n},k=0,1,2,\cdots \;,n,0<\tau <p$. As shown in Table 1, number 1 is the distributed control protocol in the case of no time-delay, it can be found that the upper limit of the sampling period depends on the eigenvalue of $\bar{L}$ through the control protocol (11) proposed by Theorem 1. For the case with time delay is number 2, by applying control protocol (21) and observation Theorem 2, we can find that the upper limit of time-delay τ depends on the eigenvalue of $\bar{L}$, and the sampling period is not only related to the eigenvalue of $\bar{L}$ but also related to the value of τ. This means that on the premise that τ meets the value condition, the upper limit of sampling period *p* can be further calculated, and to obtain the corresponding value range of sampling period *p* under different time-delay τ values.

**Table 1:** Comparison of conditions for achieving average consensus in distributed control protocols.

**Number**	**Distributed control protocols**	**Conditions**
1	$u_{i}\left(t\right)=\sum_{j\in N\left(i\right)}{\bar{a}}_{ij}\left(x_{j}\left(kp\right)-x_{i}\left(kp\right)\right),t\in \left[kp,kp+p\right)$	$0<p<\text{min}\frac{2}{\lambda_{i}}$
2	$u_{i}\left(t\right)=\{\begin{array}{l} \underset{j\in N\left(i\right)}{\sum }{\bar{a}}_{ij}\left(x_{j}\left(kp-p\right)-x_{i}\left(kp-p\right)\right),t\in \left[kp,kp+\tau \right)\\ \underset{j\in N\left(i\right)}{\sum }{\bar{a}}_{ij}\left(x_{j}\left(kp\right)-x_{i}\left(kp\right)\right),t\in \left[kp+\tau ,kp+p\right) \end{array}$	$\begin{array}{c} \;\tau <\text{min}\frac{1}{\lambda_{i}}\\ 0<p<\text{min}\frac{2}{\lambda_{i}}+2\tau \end{array}$

## 5. Simulations

**Figure 1:**
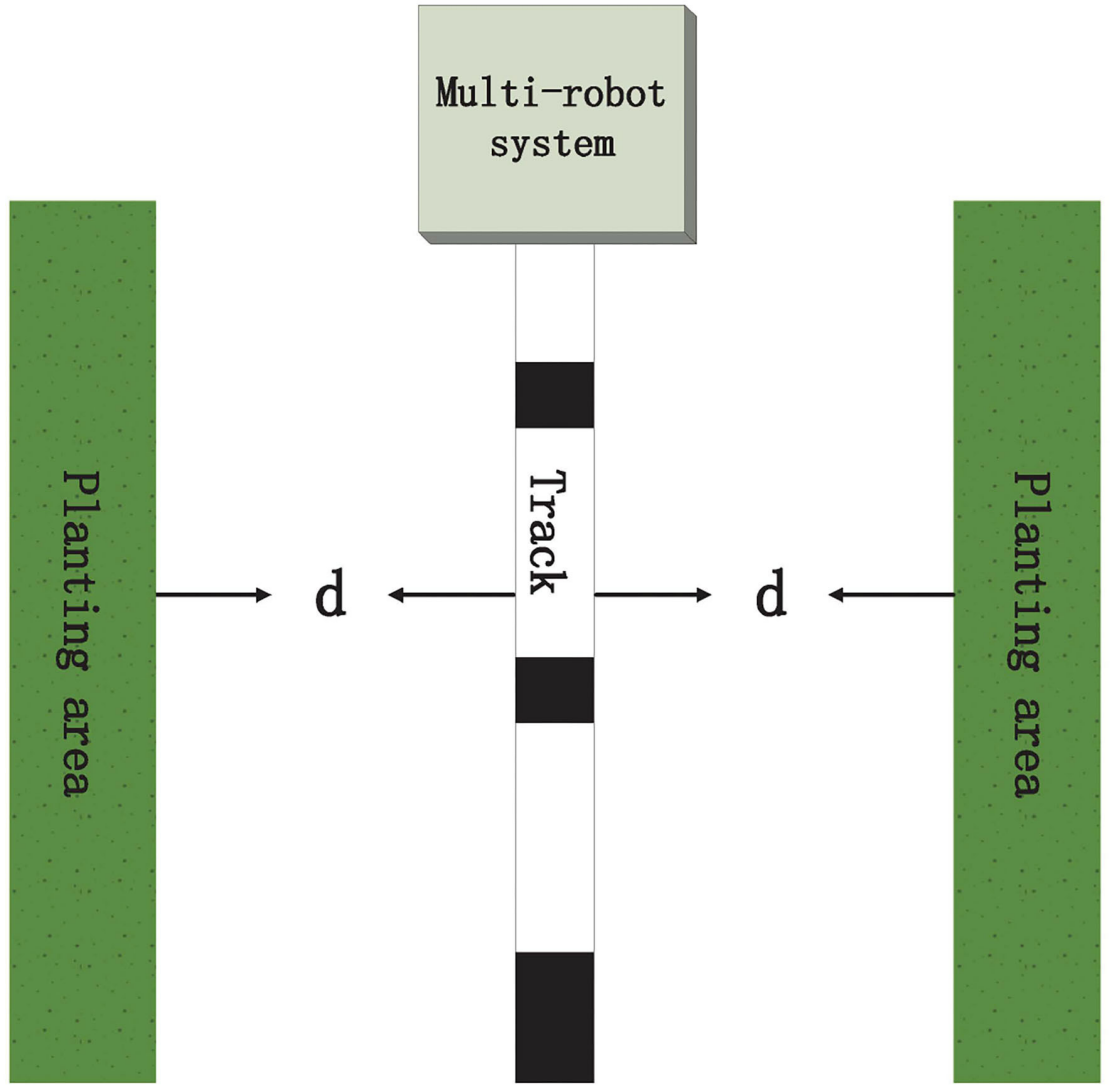
Agriculture picking multi-robot system.

This section will introduce two simulations to illustrate the correctness of our developed theoretical results. Here, we consider agriculture picking a multi-robot system including six robots in Figure 1, in which robots can pick the vegetables along the track. From Figure 1, we can see that the mechanical arm length of multi-robots needs to satisfy the distance between multi-robots and two plant areas for the purpose of conveniently gathering the vegetables. Hence, we should select a suitable orbital position to meet the distance *d*. Without loss of generality, we assume that the position state of the orbit is 5 such that the distance between orbit and planting area is *d*. Motivated by distributed averaging, we can pick up the initial states of six robots as follows:


$$\begin{array}{l} x\left(0\right)={\left[2,6,7,3,4,8\right]}^{T}. \end{array}$$

**Figure 2:**
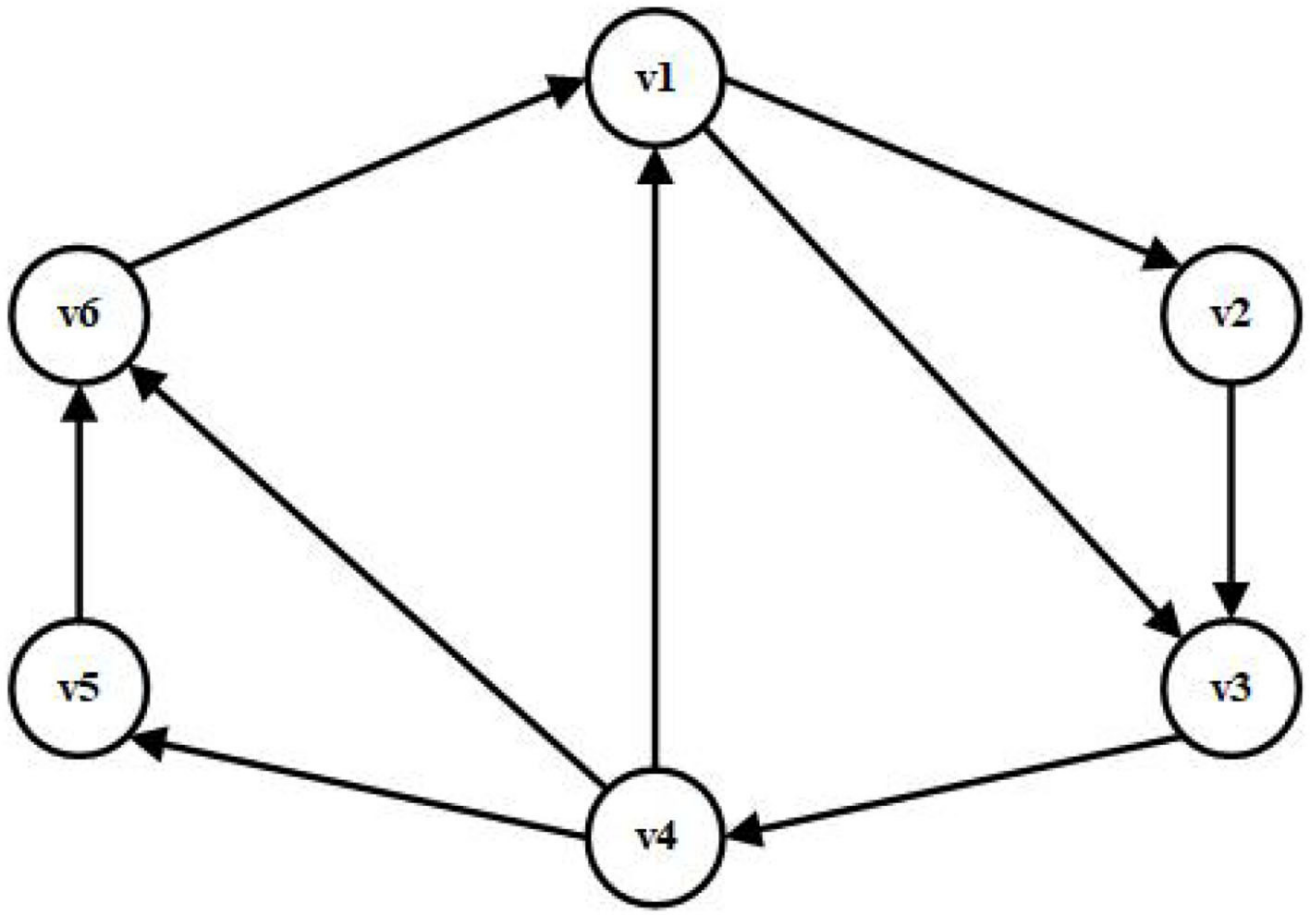
Strong connected digraph $G_{1}$.

**Example 1**. The communication topology of multi-robots can be described in Figure 2. We can easily see from Figure 2 that the digraph $G_{1}$ is strongly connected and weight unbalanced, in which only the weight 1 is considered. We can get the Laplacian matrix *L* of $G_{1}$. By Lemma 1, the specific values of the matrix $\bar{L}$ are computed, and the eigenvalues of $\bar{L}$ are further obtained. With the help of Theorem 1, then we can compute the upper bound value of *p* is 0.16198. Hence, the system (12) can reach the consensus objective if and only if *p* < 0.16198 holds.

**Figure 3:**
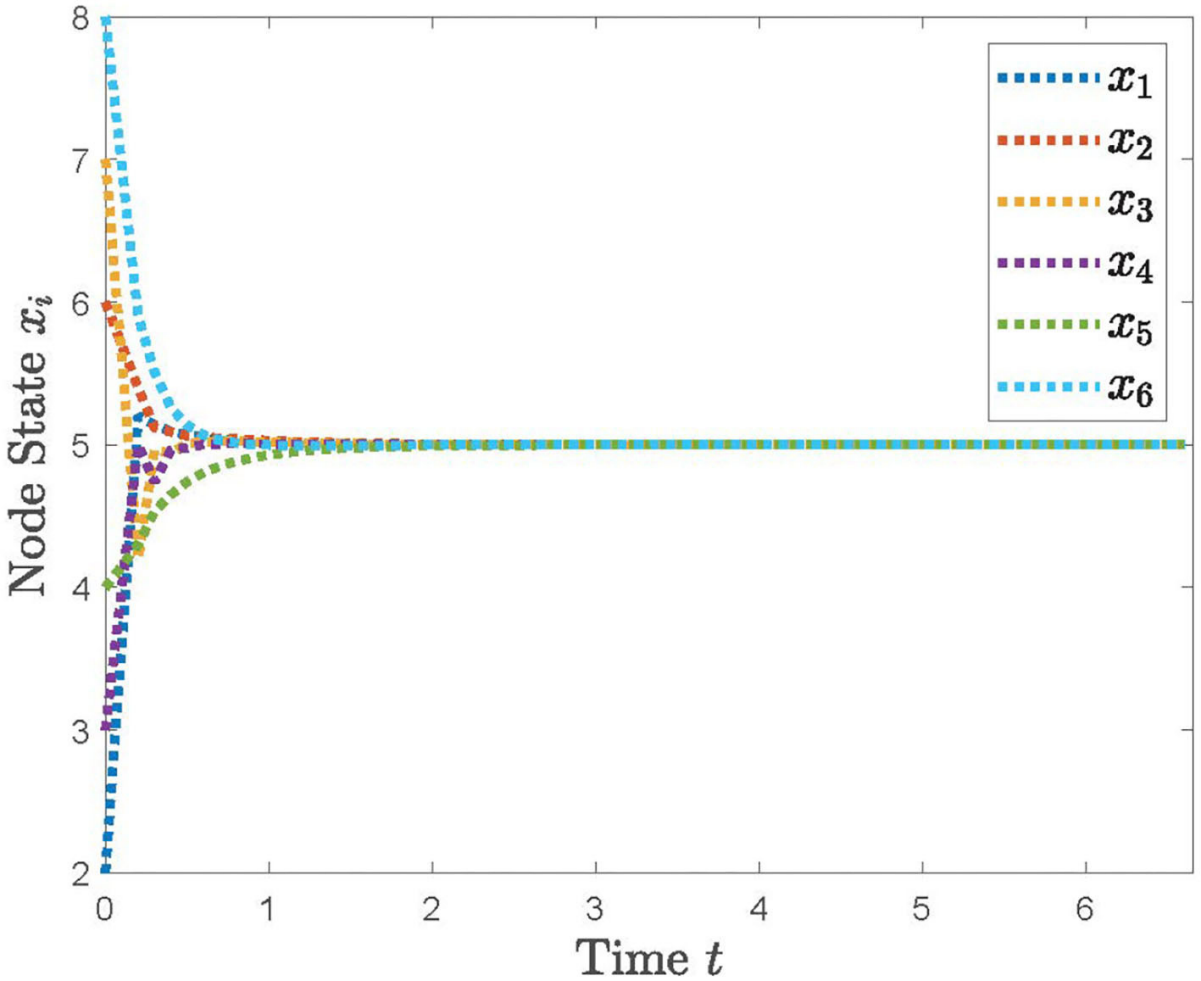
The simulation result with *p* = 0.1.

**Figure 4:**
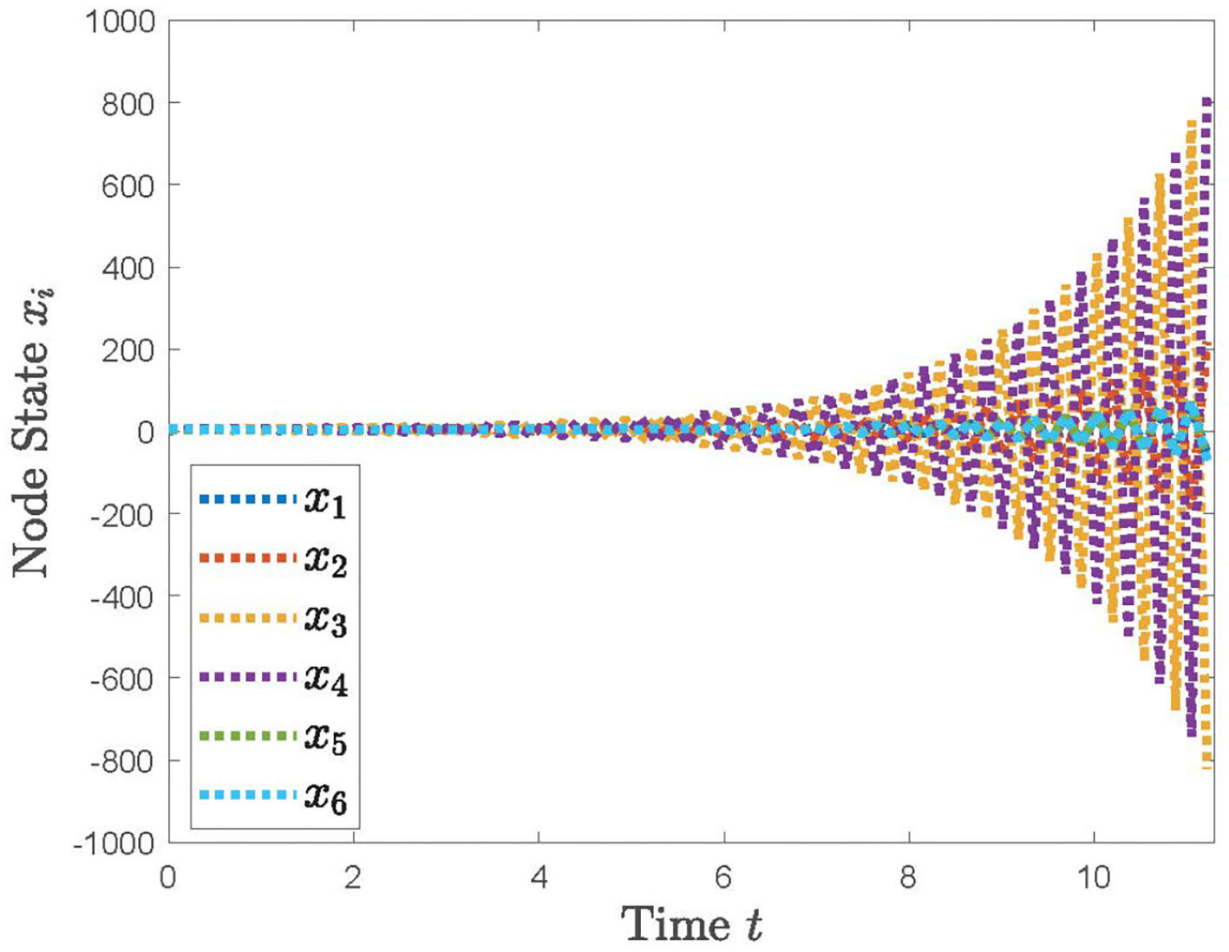
The simulation result with *p* = 0.17.

With the distributed control protocol (11) being employed, the state evolution of the system (12) with the sampled period *p* = 0.1 is plotted in Figure 3 and with the sampled period *p* = 0.17 in Figure 4. It is obvious from Figures 3, 4 that the system (12) can achieve the average consensus objective with value 5 when the sampled instant is *p* = 0.1 and the system (12) is divergent when the sampled instant is *p* = 0.17, which coincide with the results of Theorem 1. Therefore, our designed protocol can ensure the multi-robots reach the arbitrary expected position by selecting the initial states of the robots.

**Figure 5:**
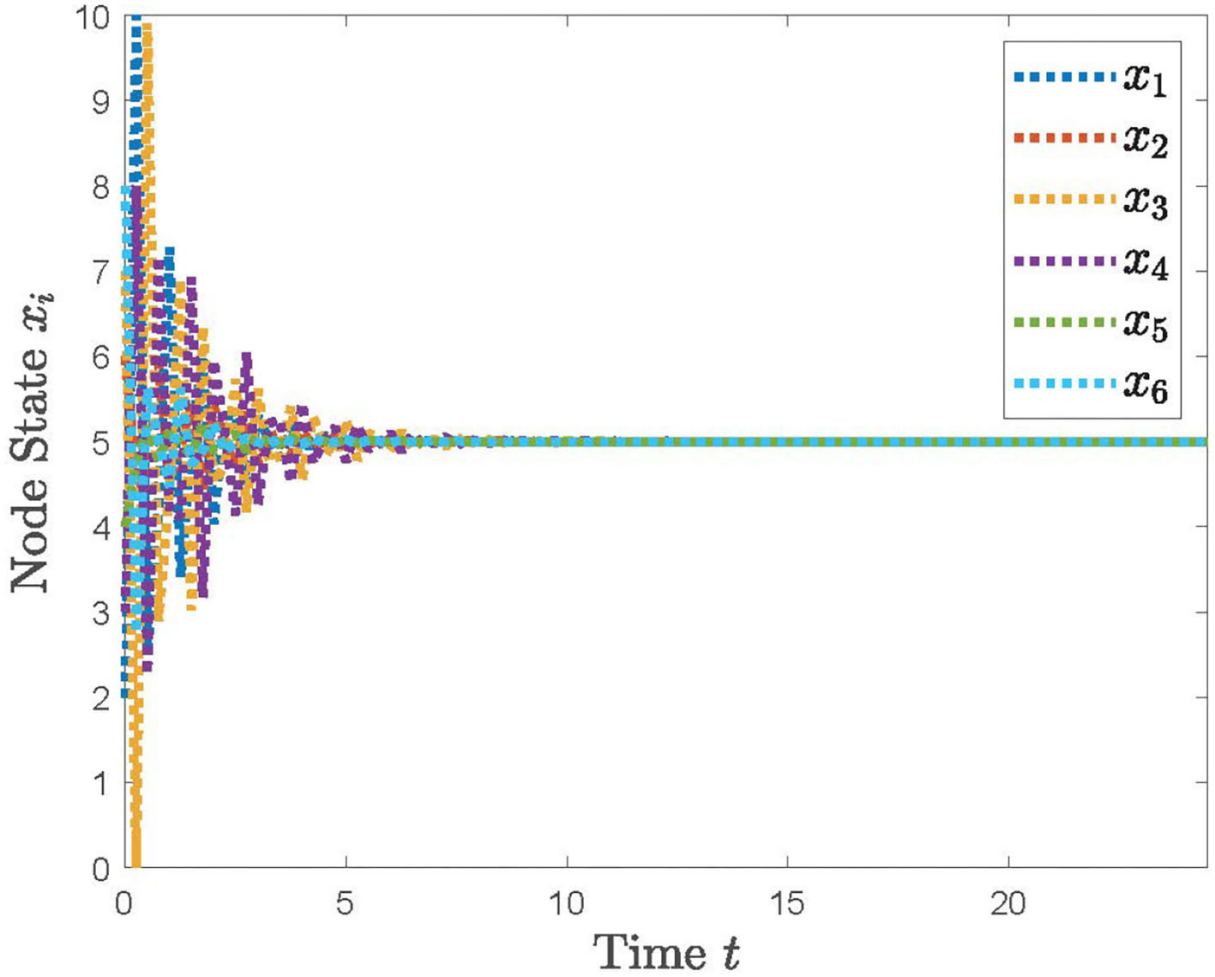
The simulation result with τ = 0.06, *p* = 0.25.

**Figure 6:**
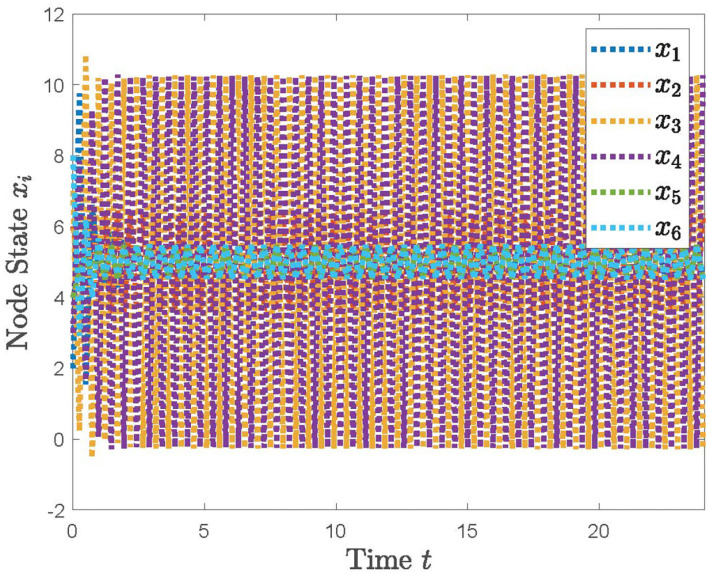
The simulation result with τ = 0.04, *p* = 0.24198.

**Figure 7:**
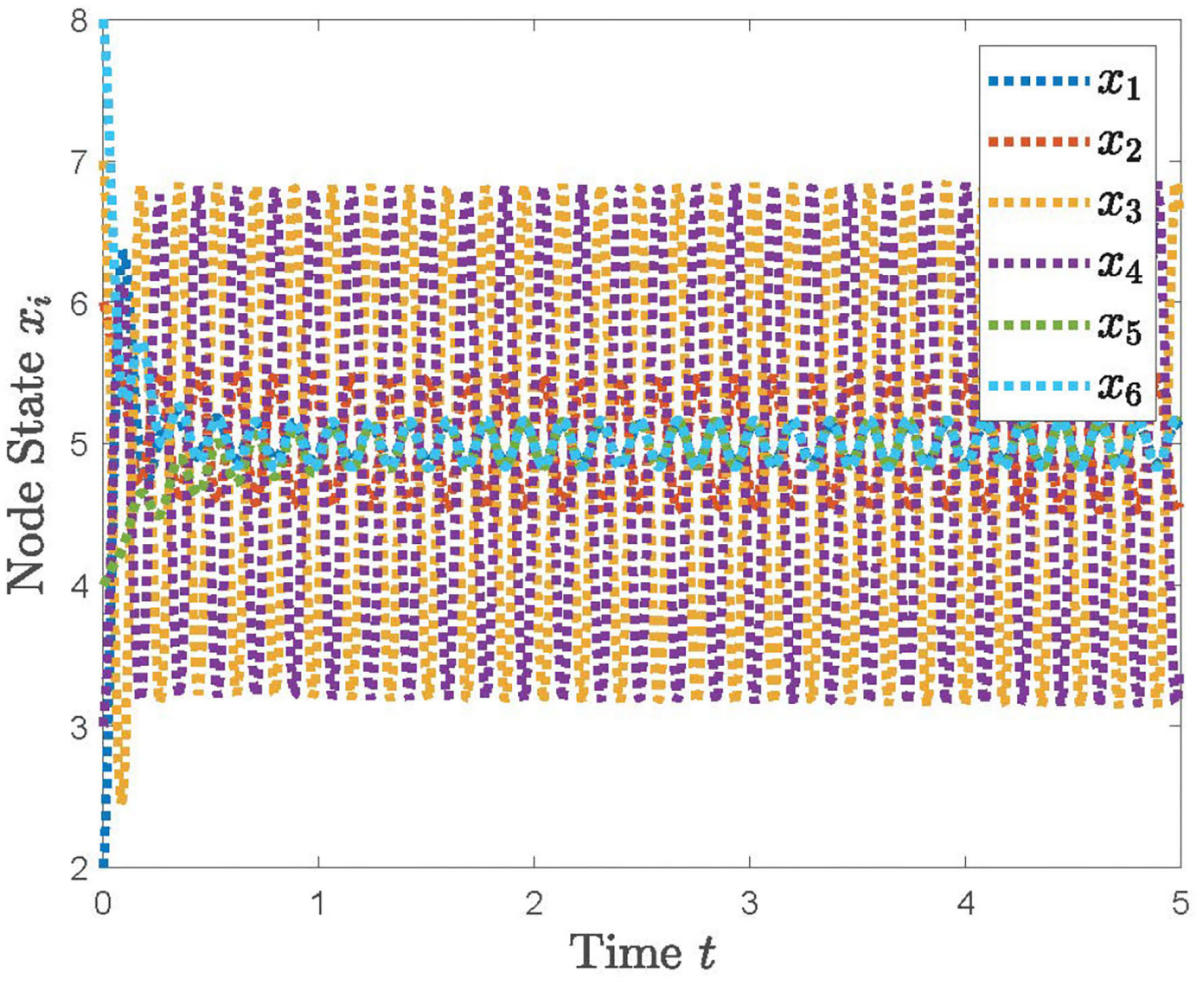
The simulation result with τ = 0.081, *p* = 0.3.

**Example 2**. When considering the communication time-delay that is inevitable in communication among agents, we can calculate that the time margin τ satisfies τ_*max*_ = 0.081 and $p<\frac{2}{\lambda_{max}}+2\tau$ based on (28). The state evolution of the system (22) with τ = 0.06, *p* = 0.25, τ = 0.04, *p* = 0.24198, and τ = 0.081, *p* = 0.3 are plotted in Figures 5–7, respectively. From Figures 5–7, we can see that the system (12) can accomplish the average consensus objective if τ_*max*_ = 0.081 and $p<\frac{2}{\lambda_{max}}+2\tau$ hold and diverge, otherwise. Hence, the effectiveness of Theorem 2 can be verified.

## 6. Conclusion

To prevent information transfer redundancy and reduce the cost of the agricultural multi-agent systems, this article has investigated the distributed averaging problems of directed agriculture picking multi-robot systems with sampling control. We have designed the distributed control protocols with and without time-delay by neighbor information to accomplish the average consensus objective, respectively. It is shown that the necessary and sufficient conditions have been provided for the average consensus, and the robots of the agriculture picking multi-robot system can achieve the purpose of consensus position state by setting the initial value. Two simulation examples have been introduced to demonstrate the effectiveness of our derived results. The distributed control protocols designed by sampling control can achieve the average consensus, which means that the agents of smart agricultural multi-robot systems can achieve state consensus, and the robots of agricultural multi-robot systems can accurately collect vegetables picked by mechanical arms. The protocols we designed also can reduce the information redundancy and control costs of smart agricultural multi-agent systems by sampling control, even agricultural multi-agent systems with time-delay, and provide a feasible method to improve the mechanization level of smart agriculture. At present, the proposed protocols are only applicable to the structure proposed, and only apply to the case where the sampling period is fixed, but we have further studied whether it is still applicable in other environments. In the future, we attempt to change the problem of the fixed sampling period into the study of the variable sampling period, so that the multi-robot systems can achieve average consistency under more working conditions and further promote the smooth completion of cooperative tasks among robots.

## Data Availability Statement

The original contributions presented in the study are included in the article/supplementary material, further inquiries can be directed to the corresponding author/s.

## Author Contributions

FM: funding acquisition and project administration. HY: writing—original draft. MD: conceptualization, methodology, and writing—reviewing and editing. PJ: supervision. XS: software. All the authors have read and agreed to the published version of the manuscript.

## Funding

This study was supported in part by the National Natural Science Foundation of China under Grants 62103210 and 61903207, in part by the Industry-university-research Collaborative Innovation Fund project of Qilu University of Technology (Shandong Academy of Sciences) under Grant 2020-CXY26, and in part by the Key Technology Research and Development Program of Shandong (Major Scientific and Technological Innovation Project) under Grants 2019JZZY010731 and 2020CXGC010901.

## Conflict of Interest

The authors declare that the research was conducted in the absence of any commercial or financial relationships that could be construed as a potential conflict of interest.

## Publisher's Note

All claims expressed in this article are solely those of the authors and do not necessarily represent those of their affiliated organizations, or those of the publisher, the editors and the reviewers. Any product that may be evaluated in this article, or claim that may be made by its manufacturer, is not guaranteed or endorsed by the publisher.
